# Integrated Positioning System of Kiwifruit Orchard Mobile Robot Based on UWB/LiDAR/ODOM

**DOI:** 10.3390/s23177570

**Published:** 2023-08-31

**Authors:** Liangsheng Jia, Yinchu Wang, Li Ma, Zhi He, Zixu Li, Yongjie Cui

**Affiliations:** 1 College of Mechanical and Electrical Engineering, Northwest A&F University, Xianyang 712100, China; 2 Key Laboratory of Agricultural Internet of Things, Ministry of Agriculture and Rural Affairs, Xianyang 712100, China; 3 Shaanxi Key Laboratory of Agricultural Information Perception and Intelligent Service, Xianyang 712100, China

**Keywords:** mobile robot in kiwifruit orchards, outdoor integrated positioning, UWB positioning, Kalman filtering, particle filtering

## Abstract

To address the issue of low positioning accuracy of mobile robots in trellis kiwifruit orchards with weak signal environments, this study investigated an outdoor integrated positioning method based on ultra-wideband (UWB), light detection and ranging (LiDAR), and odometry (ODOM). Firstly, a dynamic error correction strategy using the Kalman filter (KF) was proposed to enhance the dynamic positioning accuracy of UWB. Secondly, the particle filter algorithm (PF) was employed to fuse UWB/ODOM/LiDAR measurements, resulting in an extended Kalman filter (EKF) measurement value. Meanwhile, the odometry value served as the predicted value in the EKF. Finally, the predicted and measured values were fused through the EKF to estimate the robot’s pose. Simulation results demonstrated that the UWB/ODOM/LiDAR integrated positioning method achieved a mean lateral error of 0.076 m and a root mean square error (RMSE) of 0.098 m. Field tests revealed that compared to standalone UWB positioning, UWB-based KF positioning, and LiDAR/ODOM integrated positioning methods, the proposed approach improved the positioning accuracy by 64.8%, 13.8%, and 38.3%, respectively. Therefore, the proposed integrated positioning method exhibits promising positioning performance in trellis kiwifruit orchards with potential applicability to other orchard environments.

## 1. Introduction

Currently, the positioning and navigation methods for orchard robots worldwide primarily include Global Navigation Satellite Systems (GNSS) navigation, machine vision navigation, LiDAR navigation, and multi-sensor fusion navigation [1,2,3,4]. GNSS navigation is widely employed in open-field agricultural operations due to its real-time provision of absolute positioning information, high precision, and all-weather capability [5,6]. However, the stable reception of satellite signals by GNSS is obstructed in the environment of trellis-style kiwifruit orchards due to shielding from tree leaves and signal interference from the metallic trellis wires [7,8]. Consequently, the applicability of GNSS navigation becomes limited, and it cannot accurately fulfill navigation tasks.

With the advancement of image technology and decreasing economic costs, vision sensors are extensively applied in the field of agricultural robots because of their rich information content and low cost [9]. Benson et al. [10] developed a machine vision system to guide a combine harvester, which utilizes the lateral position of crop cutting edges as a guidance reference. Using convolutional layer feature visualization techniques, Gao et al. [11] investigated the influence of the depth of convolutional neural networks on the feature extraction of kiwifruit tree trunks. They focused on extracting features at the connection between the tree trunk and the furrow, fitting a navigation line based on the detected kiwifruit tree trunk targets. The average lateral deviation was 7.15 cm under black furrow conditions, 6.29 cm under inter-row grass conditions, and 7.36 cm under plastic film-covered conditions. Although visual navigation offers many advantages, it is easily affected by lighting conditions and thus, cannot meet navigation requirements in outdoor environments [12,13]. Additionally, due to the complex environment of orchards, image information loss can occur, affecting the accuracy of the navigation system. Foliage, branches, and weeds in orchards can potentially obstruct the camera’s line of sight, resulting in the loss of image information in certain areas [14]. Fruits, leaves, bare soil, shadows, and background environments in orchards can introduce color confusion, thereby affecting the color recognition capabilities of the visual system [15]. Adverse weather conditions such as rain, fog, frost, or snow can degrade the performance of the visual system, leading to blurry and indistinguishable images [16].

Laser navigation has numerous advantages, such as high-ranging accuracy, good resolution, and strong anti-interference capabilities, and it is widely applied in orchard environment perception [17]. Jones et al. [18] designed an autonomous navigation heavy-duty platform for kiwifruit orchards based on multi-line laser radar. Thanpattranon et al. [19] utilized a 2D laser rangefinder and employed a Kubota Kingwel KL-21 tractor as the experimental platform to develop an automatic curve navigation system suitable for orchards. The experimental results showed an average path tracking deviation of 0.275 m with a standard deviation of 0.009 m. However, drift occurred after a period of operation, resulting in divergence in positioning and accumulating errors. Therefore, a positioning method that provides absolute positioning information is needed to address this issue.

Xie et al. [20] proposed an improved angle-of-arrival (AOA) model for agricultural machinery navigation parameter detection based on UWB base station tag relative ranging information. This method realized cost-effective, high-precision, and simple autonomous navigation in field environments. By using UWB as the sole source of positioning information, this method faces challenges in providing highly continuous and stable positioning information.

The single-sensor approach has inherent limitations and exhibits poor navigation stability in complex and dynamic environments [21,22,23]. The development of the Bin-Dog orchard transport robot by Washington State University in the United States addressed this issue. The robot was equipped with sensors such as GPS and laser scanners, enabling efficient handling tasks in orchard inter-rows and field turning functions [24]. Kanagasinghamd et al. [25] integrated GNSS, compass, and machine vision into a rice field weeding robot, achieving fully autonomous navigation for weed control operations. The proposed system demonstrated excellent performance in low weed density conditions, with heading compensation accuracy below 2.5° and an average deviation from the ideal path of 45.9 mm. Gao et al. [26] utilized the complementary characteristics of Global Positioning System (GPS) and LiDAR to periodically calibrate the Inertial Navigation System (INS) under different environmental conditions. Real experiments were conducted on unmanned ground vehicles (UGV) in both outdoor and indoor environments. The results demonstrated sub-meter navigation accuracy throughout the entire trajectory. Jaeger-Hansen et al. [27] estimated the position of grapefruit trees in a citrus orchard using GNSS and LiDAR for autonomous robot mission planning and positioning. Experimental results showed an average accuracy of 0.2 m for estimating positions along the centerline and 0.35 m in the direction perpendicular to the rows. Guevara et al. [28] fused information derived from LiDAR scan matching estimation with GNSS measurements to reduce errors associated with GNSS receivers. Testing was conducted in an apple orchard, and the results showed a 20% reduction in estimation errors for crown surface area, crown volume, and porosity when the GNSS error was 1.2 m, with even greater reductions of 50% for smaller errors.

During autonomous navigation of robots in orchards, sensor data may be affected by noise due to conditions such as the soft and uneven ground of the orchard. To mitigate this impact, navigation data processing methods based on filtering technology can be used to filter and reduce the noise in the data. When using multi-sensor fusion, it is also necessary to analyze and process redundant or complementary information in the data to achieve an optimal estimate of the robot’s surrounding environment and its own state. Commonly used navigation data processing methods based on filtering include KF, PF, and EKF [29]. These methods help improve the accuracy and reliability of the data obtained by the robot. The KF is a linear filtering and prediction method that provides a robust mathematical method for real-time multi-sensor fusion and noise reduction. By inputting and outputting observation data, the state of the system can be optimally estimated. Since mobile robot systems are mostly non-linear, the EKF method is used to solve the non-linear system problems of mobile robots. EKF linearizes non-linear systems through the Taylor expansion of non-linear functions and then operates as a regular KF. Tang et al. [30] proposed a differential adaptive and EKF combined algorithm and verified its effectiveness. The PF is a non-linear filtering method that combines Bayesian with Monte Carlo random sampling methods and is not constrained by the assumptions of linear systems and Gaussian noise. Jie Ying et al. [31] applied the PF method to multi-sensor data fusion and added a step to resist outliers in the algorithm, effectively mitigating the error caused by GPS jumps, and thus obtaining accurate navigation and positioning information. Compared with traditional filtering methods, Rao–Blackwellized Particle Filters (RBPF) and Rao–Blackwellized Kalman Filters (RBKF) represent advanced techniques in the field of state estimation. They overcome the limitations of traditional filtering methods, providing more accurate and efficient state estimation methods, especially suitable for complex and nonlinear systems. RBPF and RBKF combine the concepts of PF and KF, where certain state variables can be efficiently estimated using KF, while other variables employ PF. This decomposition and combination approach can effectively reduce computational complexity while providing more precise state estimation, particularly in high-dimensional and non-linear systems [32]. Gupta et al. employed RBPF for the fusion of GNSS and visual odometer, which combines the tracking efficiency of KF with the superior uncertainty modeling of PF, enabling effective state tracking and rich position probability distribution [33]. Norhidayah et al. adopted RBPF in a grid-based Simultaneous Localization and Mapping (SLAM) algorithm, effectively improving the mapping accuracy of the map and significantly reducing the error in robot state estimation [34]. The integrated positioning method in this study is also implemented by combining KF, EKF, and PF, fully utilizing the advantages of each individual method to improve the accuracy of robot positioning.

In summary, the fusion of multiple sensors generally leverages the advantages of continuous relative positioning information provided by the sensors and the absence of cumulative errors in absolute positioning information, leading to high accuracy. In outdoor environments, GNSS is commonly used to obtain the robot’s absolute positioning information. However, in a trellis-style kiwifruit orchard environment, the presence of overhead structures obstructs GNSS signals, and the use of LiDAR for robot positioning and navigation can result in accumulated odometry errors due to track slippage and prolonged travel. On the other hand, UWB positioning can provide high-precision positioning information without cumulative errors and with real-time capabilities even in GNSS-denied environments. Therefore, this study conducts research on the integrated positioning method of UWB/LiDAR/ODOM in a trellis-style kiwifruit orchard environment to correct the robot’s cumulative positioning errors and improve the accuracy of its positioning and navigation.

## 2. Materials and Methods

### 2.1. System Components

**Figure 1:**
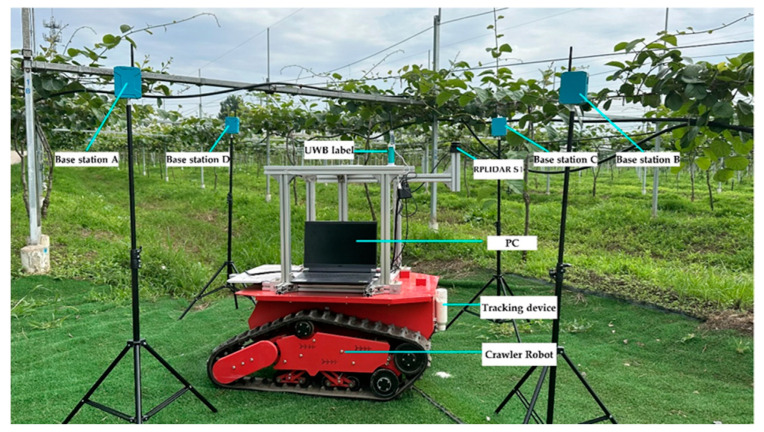
Diagram of the integrated positioning system.

**Figure 2:**
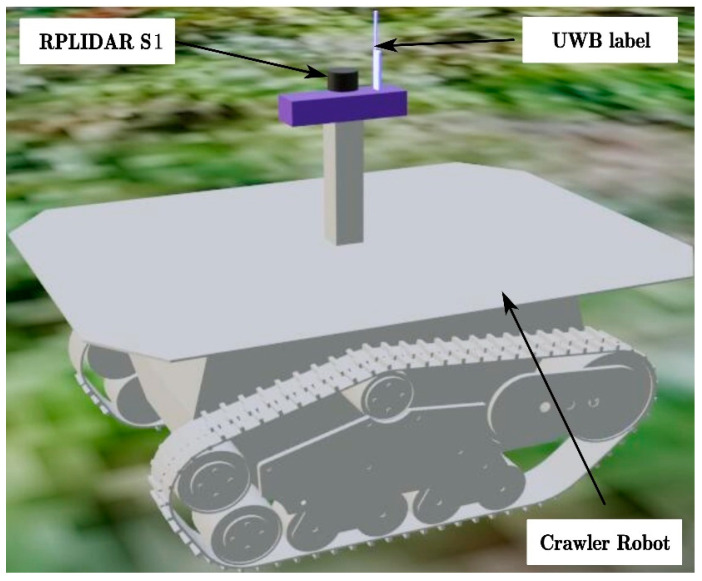
Gazebo model of the integrated positioning system.

As shown in Figure 1, the integrated positioning system of this study comprises primarily a crawler robot, a UWB tag, a LiDAR sensor, a personal computer (PC), and a tracking device. In addition, the Gazebo model of the integrated positioning system is depicted in Figure 2. The crawler robot operates on a two-wheel differential mode, with each track unit including an independent active wheel. It has a length of 1020 mm and a width of 790 mm. The passive wheel primarily supports the robot’s movement in various directions. The detailed specifications of the sensors and motors used in this study are available in Appendix A Table A1. The LiDAR sensor used is the RPLiDAR S1 model. To match the actual specifications of RPLiDAR S1, the parameters of the simulation model are consistent with the actual LiDAR parameters. UWB is an absolute positioning method that calculates distance by measuring signal propagation time, and its ranging accuracy directly affects positioning accuracy. Its precision is influenced by the orchard’s obstacles and the sensor’s noise level. For UWB simulation modeling, we developed a UWB positioning feature pack in ROS, based on the DS-TWR ranging principle. To match the actual specifications of the D-DWG-PGPLUS positioning module, we simulated different complex environments’ effects on UWB sensor positioning accuracy by adding an offset error to the tag to base station ranging distance in the simulation model parameters. We modeled the UWB sensor’s internal noise by having the noise in the tag-to-base station ranging distance follow a Gaussian distribution with a standard deviation of 0.1 m and 0.3 m. The simulated UWB has an update rate of 10 Hz. Two photoelectric encoders, installed separately on the two motor drive wheels, provide speed and mileage information as ODOM. The odometer is used for the relative positioning of the robot, and its accuracy is affected by ground conditions and sensor noise. Inaccurate odometer readings can lead to cumulative errors, thereby affecting positional accuracy. For ODOM simulation, the robot’s odometer is calculated based on the active wheel’s speed on both sides. To match the encoder’s real specifications, noise is added to the speeds of both wheels, making the noise follow a Gaussian distribution with a standard deviation of 0.05 m. To replicate the ground conditions of kiwifruit orchards, we used the SketchUp sandbox tool to create the terrain, incorporating pyramid-shaped protrusions randomly into a flat grid to simulate bumps. The simulated ODOM has an update rate of 10 Hz. In the simulation process of automatic navigation for the robot within Gazebo, a synergistic relationship between Gazebo and the Robot Operating System (ROS) facilitates the interaction between the simulated environment and control algorithms. Within Gazebo, sensors generate data through simulation, mimicking data gathered by actual sensors in reality. The communication between Gazebo and ROS is established using plugins provided by ROS. These plugins can simulate various sensors with parameters corresponding to each sensor set within the plugin and sensor models, such as noise, resolution, etc., endeavoring to emulate the behavior of real sensors as closely as possible. Within the ROS framework, proprietary navigation algorithms can be developed, encompassing multi-sensor combined positioning algorithms, Simultaneous Localization and Mapping (SLAM) algorithms, and path planning algorithms, among others. These algorithms subscribe to sensor data and publish control directives, facilitating navigation of the robot within the simulation environment. Control instructions are conveyed to Gazebo via ROS topics, dictating the motion of the robot. Upon receipt of control directives published by ROS, Gazebo updates the simulated state considering the robot model alongside physical properties and environmental presets, resulting in a simulated movement and navigation of the robot within the virtual world. The crawler robot uses the STM32 module as a lower machine to control the DC motor driving the active wheel, monitoring, and feedback on the travel speed of the mobile platform through the photoelectric encoder. The crawler robot uses a personal computer (PC) with Ubuntu (16.04) and ROS (Kinetic) systems installed as the upper machine. Based on ROS (Kinetic), this platform achieves functions such as positioning navigation algorithms, sensor data monitoring, and issuing speed control instructions [35,36]. The tracking device contains white flour, which leaves a white line on the ground when the robot moves, representing the actual path taken by the robot.

### 2.2. Simulation Environment Setup

**Figure 3:**
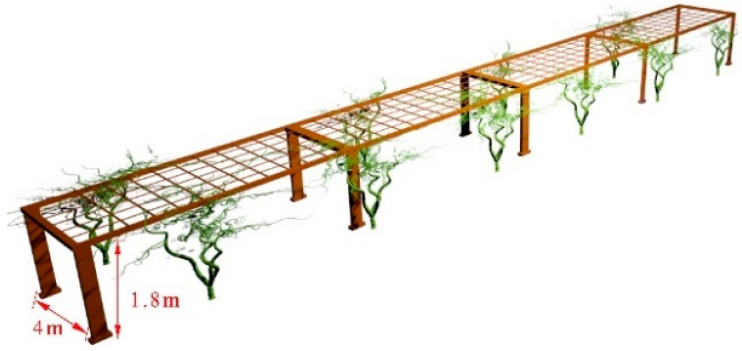
Individual trellis simulation model.

**Figure 4:**
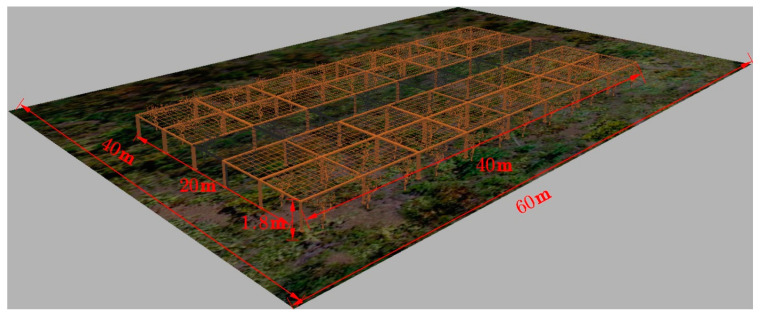
Kiwifruit orchard simulation environment.

We created a highly realistic model of a trellis-style kiwifruit orchard in SketchUp and imported it as a 3D model file into Gazebo. The obstacles, terrain, and physical properties in the created simulated environment model of the kiwifruit orchard are based on an actual trellis-style kiwifruit orchard. Unlike the real environment, natural conditions such as wind, rain, snow, and moving obstacles were not taken into consideration in our model. Each trellis is 4 m wide and 1.8 m high, as shown in Figure 3. As depicted in Figure 4, the entire trellis measures 20 m in width and 40 m in length. To simulate leaf coverage and density, kiwifruit trees are randomly distributed on both sides and exhibit around 50% overlap of branches and leaves. The soil type of the kiwifruit orchard is primarily loamy soil and sandy soil, covered with weeds, with ground protrusions less than 5 cm [37,38]. To replicate the actual environment, we used the SketchUp sandbox tool to construct the ground and create a test area of 40 × 60 m, as shown in Figure 4. By randomly creating pyramid-shaped protrusions in the flat grid, bumps were simulated, with heights of both 2 cm and 4 cm.

### 2.3. Positioning Method

**Figure 5:**
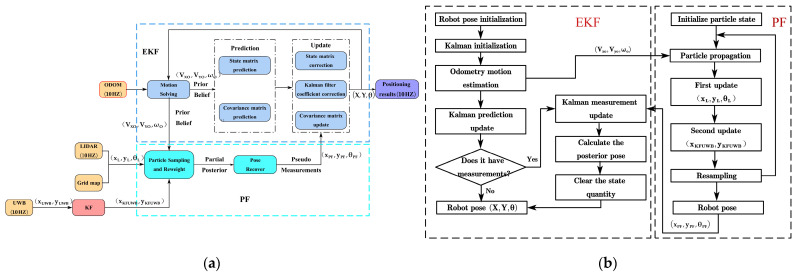
Integrated positioning methods. (**a**) Integrated positioning framework diagram. (**b**) Synergistic application of PF and EKF.

The integrated positioning method proposed in this study is illustrated in Figure 5. First, a dynamic error correction method based on Kalman filtering is applied to achieve accurate positioning of the UWB sensor and reduce the dynamic positioning error. Second, the filtered UWB, LiDAR measurements, and ODOM values are fused using particle filtering, where the particle-filtered robot pose is utilized as the measurement for the EKF, while the ODOM measurement serves as the prediction for the EKF. Finally, the predicted and measured values are fused using extended Kalman filtering to estimate the robot’s pose. In describing the integrated positioning method, we have used a series of variables. Their definitions and meanings can be found in the table in the Appendix A, specifically see Table A2 in the Appendix A.

The specific steps of the proposed integrated positioning algorithm are as follows (Algorithm 1):
**Algorithm 1** Specific steps of the proposed integrated positioning algorithm  Step 1: Input the robot’s position $\left(x_{UWB},y_{UWB}\right)$ measured by UWB, the pose $\left(x_{L},y_{L},\theta_{L}\right)$ obtained by LiDAR scanning, and the values $\left(V_{xo},V_{yo},\omega_{o}\right)$ from the odometer.  Step 2: Apply a KF (refer to Section 2.3.1) to the robot’s position $\left(x_{UWB},y_{UWB}\right)$ measured by UWB.  Step 2.1: Determine whether the UWB measurement value is an outlier based on the outlier judgment condition proposed in this study.  Step 2.2: When the UWB measurement is found to be an outlier, apply the KF model proposed in this study for filtering, or proceed to Step 2.5.  Step 2.3: Use the robot’s positional at time $t_{1}$, $t_{2}$ to predict the position at time $t_{3}$.  Step 2.4: Use the UWB measurement at time $t_{3}$ to update the predicted pose.  Step 2.5: Output the measurement value $\left(x_{KFUWB},y_{KFUWB}\right)$ after applying the KF.  Step 3: Perform PF fusion (refer to Section 2.3.2) on the measurement value $\left(x_{KFUWB},y_{KFUWB}\right)$ from the KF, the robot’s pose $\left(x_{L},y_{L},\theta_{L}\right)$ obtained by LiDAR scanning, and the odometer measurement $\left(V_{xo},V_{yo},\omega_{o}\right)$.  Step 3.1: Use the odometer reading $\left(V_{xo},V_{yo},\omega_{o}\right)$ to predict the pose.  Step 3.2: Use the LiDAR measurement $\left(x_{L},y_{L},\theta_{L}\right)$ to update the predicted pose. This is the first update.  Step 3.3: Use the measurement value $\left(x_{KFUWB},y_{KFUWB}\right)$ after Kalman filtering to update the pose again. This is the second update.  Step 3.4: Output the estimated pose $\left(x_{PF},y_{PF},\theta_{PF}\right)$ after PF fusion.  Step 4: Perform EKF fusion (refer to Section 2.3.3) on the measurement value $\left(x_{PF},y_{PF},\theta_{PF}\right)$ from the PF and the odometer reading $\left(V_{xo},V_{yo},\omega_{o}\right)$.  Step 4.1: Use the odometer reading $\left(V_{xo},V_{yo},\omega_{o}\right)$ to predict the pose.  Step 4.2: Use the pose estimation value $\left(x_{PF},y_{PF},\theta_{PF}\right)$ obtained after particle filtering to update the predicted pose.  Step 4.3: Output the final estimated pose (X,Y,θ).

The integrated positioning method proposed in this study has the following main features. Firstly, UWB is generally used for indoor scenes, but there is less related research that uses UWB devices for positioning in orchards. This study applies UWB devices to trellis-style kiwifruit orchards, which highlights the practicality of this integrated positioning method in this particular environment. Secondly, most previous research on UWB/LiDAR fusion positioning directly combines the data measured by UWB. However, UWB positioning within orchards can be interfered with by multiple factors, leading to discontinuous positioning. If the values measured by UWB are directly fused, the positioning effect might deteriorate. Therefore, this study proposes a KF model and a threshold judgment condition for the values of UWB in a trellis-style orchard environment. The values after Kalman filtering are then combined with the information measured by other sensors, thereby effectively improving the efficiency of the method. Lastly, a combined method using Kalman filters, particle filters, and extended Kalman filters is proposed. By making full use of the advantages of each individual method such as the efficiency and simplicity of the KF, the capability of particle filters to handle more complicated and non-Gaussian systems, and the ability of extended Kalman filters to deal with nonlinear issues, the overall method provides an optimized approach for multi-sensor data fusion, especially suitable for complex situations such as applications in trellis-style orchard environments.

#### 2.3.1. UWB Positioning Error Correction Based on KF

**Figure 6:**
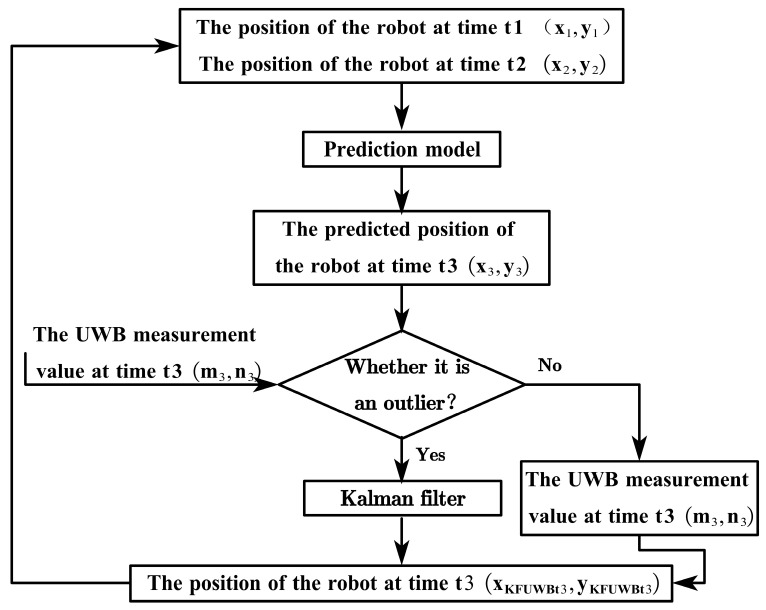
Flowchart of UWB Kalman filtering.

This study presents a dynamic error correction method based on Kalman filtering, aimed at reducing UWB positioning errors. The flowchart of the proposed method is illustrated in Figure 6.

Initially, as the robot moves, it utilizes the robot’s position information, measured by the UWB at times $t_{1}$ $\left(x_{1},y_{1}\right)$ and $t_{2}$$\left(x_{2},y_{2}\right)$, combined with the robot’s motion path, to predict the position information $\left(x_{3},y_{3}\right)$ of the robot at time $t_{3}$. The predicted location information $\left(x_{3},y_{3}\right)$ is then compared with the measurement information $\left(m_{3},n_{3}\right)$ from UWB at time $t_{3}$ and judged based on outlier determination conditions. Finally, if the measured information $\left(m_{3},n_{3}\right)$ from UWB at time $t_{3}$ is an outlier, it is merged with the predicted values $\left(x_{3},y_{3}\right)$ using KF fusion to determine the position information of the robot at time $t_{3}$. If not, we take the position $\left(m_{3},n_{3}\right)$ at time $t_{3}$ is taken as the robot’s position at time $t_{3}$.

UWB positioning relies on the range measurements between base stations and anchors, which in turn depend on the flight time of the signals [39,40]. Therefore, for UWB devices using bilateral bidirectional ranging for positioning, the flight time measurements, $T_{f}$, are consistent when the robot is stationary. However, in the case of robot motion, the time consumed for measuring the flight time, $T_{f}$, introduces a delay, causing the three measured $T_{f}$ values to not correspond to the $T_{f}$ at the same location of the target node. This discrepancy leads to positioning errors. As Kalman filtering can recursively estimate better values from the predicted and measured values using the corresponding system model [41], this study proposes a dynamic error judgment and correction method based on Kalman filtering to reduce UWB dynamic positioning errors.

**Figure 7:**
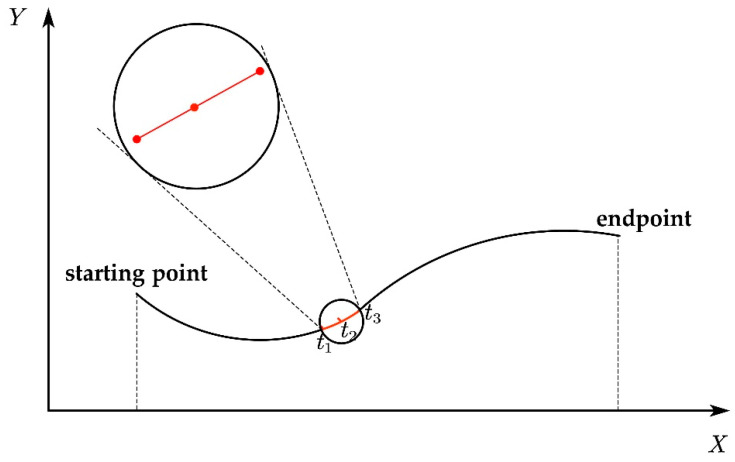
Simplified diagram of robot’s curved motion.

For instance, when a robot makes a curved movement in the orchard, the tag moves a certain distance from the starting point to the endpoint. We select the time intervals of three consecutive positions $\left[t_{1},t_{2},t_{3}\right]$, as shown in Figure 7. The black solid line represents the ground, and the larger circle is a localized magnification of the smaller circle. Here, $t_{1}$ is the first positioning time, $t_{2}$ is the second, and $t_{3}$ is the third. As the selected time is short, the tag’s trajectory from $t_{1}$ to $t_{3}$ can be seen as a straight line, and the time of each positioning is the same during the three consecutive positionings.

**Figure 8:**
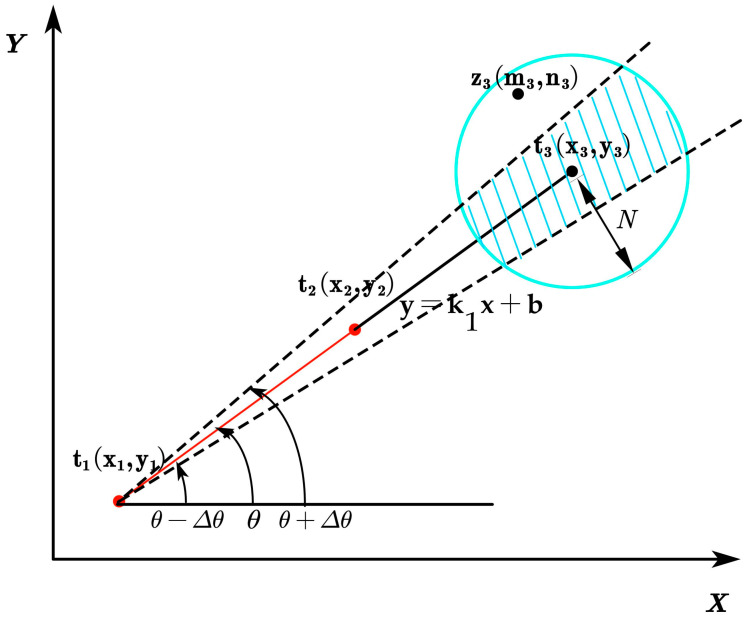
Outlier detection diagram.

Outlier judgment is shown in Figure 8. $y=k_{1}x+b$ is the line formed by the coordinates at times $t_{1}$ and $t_{2}$. The blue shaded area represents the range where normal points are located. According to the robot’s position information $\left(x_{1},y_{1}\right)$ at time $t_{1}$ and $\left(x_{2},y_{2}\right)$ at time $t_{2}$ obtained by the UWB device, we can determine the line $y=k_{1}x+b$ and the robot’s turning angle θ; then we can predict the robot’s location information $\left(x_{3},y_{3}\right)$ at time $t_{3}$.

(1) $$\left\{\begin{array}{l} x_{3}=2x_{2}\;-\;2x_{1}\\ y_{3}=2y_{2}-y_{1} \end{array}\right.$$

The data measured by the UWB positioning device at time $t_{3}$ is represented as $z_{3}$. A distance threshold N and an angle threshold Δθ are set to determine whether $z_{3}$ is an outlier.

We compare the measured value at time $t_{3}$, $\left(m_{3},n_{3}\right)$, with the predicted value at time $t_{3}$, $\left(x_{3},y_{3}\right)$.

(2) $$C=\sqrt{{\left(m_{3}-x_{3}\right)}^{2}+{\left(n_{3}-y_{3}\right)}^{2}}$$

(3) $$\Phi =\arctan((n_{3}-y_{1})/(m_{3}-x_{1}))$$

Set a threshold N as the criterion for distance judgment and a threshold Δθ as the criterion for angle change.

(4) $$\left\{\begin{array}{l} C\geq N,or\;\Phi <\theta -\Delta \theta ,or\;\Phi >\theta +\Delta \theta ,Outliers\\ C<N,and\;\theta -\Delta \theta \leq \Phi \leq \theta +\Delta \theta ,Inliers \end{array}\right.$$

Based on preliminary foundational research experiments, it has been determined that the UWB device’s average error within trellis-style kiwifruit orchards is 10 cm and the absolute value of the average angle deviation is 5 degrees. In order to provide the robot with enhanced positioning accuracy, we have set the outlier thresholds N and Δθ as 8 cm and 3 degrees, respectively. When identified as normal values, the measurement result $\left(m_{3},n_{3}\right)$ is considered as the UWB positioning result at time $t_{3}$. When identified as outliers, the outliers are corrected using the Kalman filtering method. In the Kalman filtering model used in this study, the state vector $X_{k}$ incorporates the position information at time k and k−1.

(5) $$X_{k}=\left[x_{k},y_{k},x_{k-1},y_{k-1}\right]$$

Based on the aforementioned robot motion model, the predicted position of the robot at time k can be determined.

(6) $$\left[\begin{array}{l} {\hat{x}}_{k}\\ {\hat{y}}_{k} \end{array}\right]=2\left[\begin{array}{l} x_{k-1}\\ y_{k-1} \end{array}\right]-\left[\begin{array}{l} x_{k-2}\\ y_{k-2} \end{array}\right]$$

Furthermore, the system state transition matrix A can be determined as follows.

(7) $$A=\left[\begin{array}{cccc} 2 & 0 & -1 & 0\\ 0 & 2 & 0 & 1\\ 1 & 0 & 0 & 0\\ 0 & 1 & 0 & 0 \end{array}\right]$$

In this experiment, since there are no external input control variables, the input control vector $u_{k}$ and the control matrix B at time step k are both zero. $w_{k}$ follows a Gaussian distribution with mean 0 and covariance matrix Q. Q represents the covariance of the system process, is estimated through analysis and statistical methods based on historical data. The variable $z_{k}$ is defined as $z_{k}={\left[z_{x},z_{y}\right]}^{T}$. The matrix H is responsible for transforming the state matrix $X_{k}$ into a format that can be operated with the measurement matrix $z_{k}$. Here, the matrix H is defined as follows:

(8) $$H=\left[\begin{array}{cccc} 1 & 0 & 0 & 0\\ 0 & 1 & 0 & 0 \end{array}\right]$$

$v_{k}$ follows a Gaussian distribution with mean 0 and covariance matrix R. The covariance matrix R provided by Guangzhou Networking Technology (UWB manufacturer). The Kalman algorithm can be divided into two steps: prediction and update [42,43,44]. The specific steps are as follows:

(1) Prediction:

Using the state model to predict the position:

(9) $${\hat{X}}_{k}=AX_{k-1}$$

The predicted position at time k:

(10) $${\hat{P}}_{k}=AP_{k-1}A^{T}+Q$$

(2) Update:

Calculate the Kalman gain matrix:

(11) $$K_{k}={\hat{P}}_{k}H^{T}{\left(H{\hat{P}}_{k}H^{T}+R\right)}^{-1}$$

Update the state:

(12) $$X_{k}={\hat{X}}_{k}+K_{k}(z_{k}-HX_{k-1})$$

Update the error covariance matrix:

(13) $$P_{k}=(I-K_{k}H_{k}){\hat{P}}_{k}$$

#### 2.3.2. Fusion of UWB/LiDAR/ODOM Based on Particle Filtering

**Figure 9:**
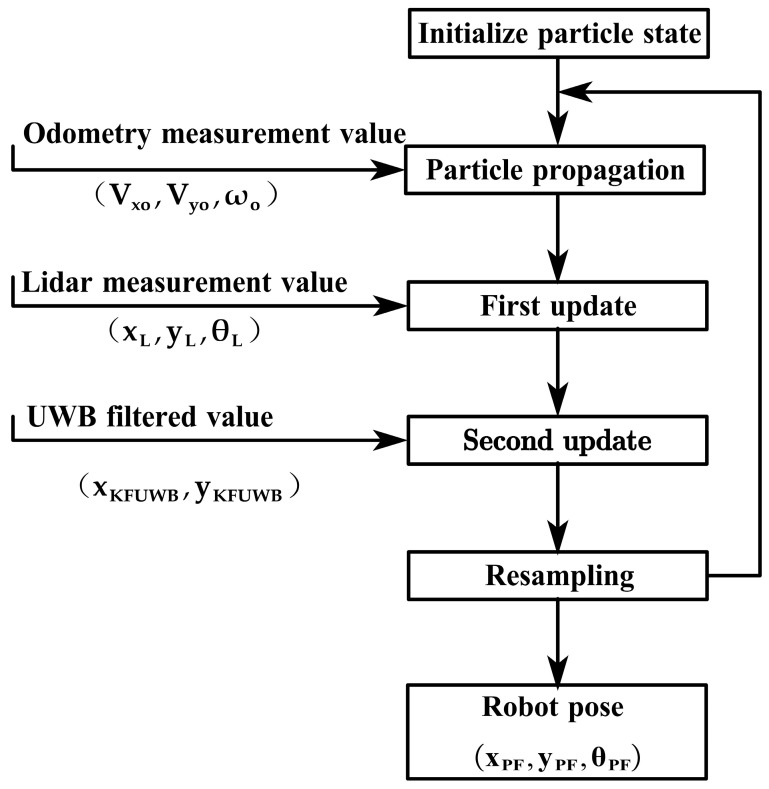
Flowchart of particle filtering.

We utilize the particle filtering approach to fuse the ODOM values, UWB position information after Kalman filtering, and LiDAR measurements [45]. The fused robot pose is then used as the pseudo-measurement for extended Kalman filtering. Particle filtering, fundamentally, is a type of Bayesian filtering that incorporates the Monte Carlo principle [46,47,48]. The goal of filtering is to obtain the posterior probability distribution of the current state. In particle filtering, the steps for updating the posterior probability are particle propagation, weight updating, and resampling [49,50]. The flowchart of the fusion algorithm is depicted in Figure 9.

Let $c_{t-1}\in C_{t-1}$ represent the particle swarm at the previous moment, $u_{t-1}=\left[V_{xt-1},V_{yt-1},\omega_{t-1}\right]$ represents the latest odometer result, and $z_{t}=[x_{Lt},y_{Lt},\theta_{Lt}]$ represents the most recent LiDAR scanning result. The goal of the algorithm is to obtain the pose ${\hat{s}}_{t}$ and particle swarm $C_{t}$ at time t.

First, we conduct the particle initialization process, which uses the particle swarm information from the previous moment to initialize the pose information $s_{t-1}$ and the particle weight information $w_{t-1}$, as shown in Equation (14). Then, we carry out particle propagation, using the odometer prediction model to obtain an estimated position value $s_{t}^{\prime i}$, as shown in Equation (15). Next, we perform the first update of the pose and weight. Based on the LiDAR observation model, a local maximum ${\hat{s}}_{t}^{i}$ is obtained through maximum likelihood estimation (MLE), as shown in Equation (16). If a local maximum is not found here, the particle’s pose state is updated using a Gaussian distribution, as indicated by Equations (17) and (18). If a local maximum is found, k poses are taken near the local maximum, as shown in Equation (19). Assuming that the k poses follow a Gaussian distribution, the mean–variance of the k poses is calculated and normalized, as depicted in Equations (20)–(24). This means that the new pose can be represented in the form of a normal distribution, as shown in Equation (25). Then, the second update of the pose is performed. The value of KFUWB is introduced to correct the mean and variance of each particle, as demonstrated in Equations (28) and (29). Finally, the current position location result is obtained through the weighted average of the particle weights and the means of each particle, as indicated in Equation (31).

(1) Particle Initialization:

Using the particle information from the previous moment to initialize pose information and particle weight information:

(14) $$<s_{t-1}^{i},\omega_{t-1}^{i}>=c_{t-1}^{i}$$

(2) Particle Propagation:

Use the odometer prediction model to obtain the estimated value of the position [51]:

(15) $${\hat{s}}_{t}^{\prime i}=s_{t-1}^{i}⊕u_{t}$$

(3) First Update:

On the basis of the LiDAR observation model, find the local maximum ${\hat{s}}_{t}^{i}$ through maximum likelihood estimation (MLE):

(16) $${\hat{s}}_{t}^{i}=\arg\max_{s}p(s|z_{t},s_{t}^{\prime (i)})$$

If no local maximum is found, a Gaussian distribution is used to update the particle’s pose state, and the observation model is used to update the particle’s weight. Then, start again from the initialization phase [52]:

(17) $$s_{t}^{\left(i\right)}∼p(s_{t}|s_{t-1}^{\left(i\right)},u_{t-1})$$

(18) $$\omega_{t}^{\left(i\right)}=\omega_{t-1}^{\left(i\right)}\cdot p(z_{t}|s_{t}^{\left(i\right)})$$

If a local maximum is found, take k poses near the local maximum:

(19) $$s_{k}∼\left\{s_{j}||s_{j}-{\hat{s}}^{\left(i\right)}|<\Delta \right\}$$

Assume that the distribution of k particles follows a Gaussian distribution, calculate their mean and normalization parameters for the k particles $s_{j}\in \left\{s_{1},\cdots ,s_{k}\right\}$ [53]:

(20) $$\mu_{t}^{\left(i\right)}=\mu_{t}^{\left(i\right)}+s_{j}•p(z_{t}|s_{j})•p(s_{t}|s_{t-1}^{\left(i\right)},u_{t-1})$$

(21) $$\eta^{\left(i\right)}=\eta^{\left(i\right)}+p(z_{t}|s_{j})•p(s_{t}|s_{t-1}^{\left(i\right)},u_{t-1})$$

Normalize the mean:

(22) $$\mu_{t}^{\left(i\right)}=\mu_{t}^{\left(i\right)}/\eta^{\left(i\right)}$$

After obtaining the mean, calculate the variance $\xi_{t}^{\left(i\right)}$ of the k particle poses [54]:

(23) $$\xi_{t}^{\left(i\right)}=\xi_{t}^{\left(i\right)}+(s_{j}-\mu^{\left(i\right)}){\left(s_{j}-\mu^{\left(i\right)}\right)}^{T}\cdot p(z_{t}|s_{j})\cdot p(s_{j}|s_{t-1}^{\left(i\right)}{,u}_{t-1})$$

Normalize the variance:

(24) $$\xi_{t}^{\left(i\right)}=\xi_{t}^{\left(i\right)}/\eta^{\left(i\right)}$$

In this way, the new pose can be represented as a normal distribution:

(25) $$s_{t}^{\left(i\right)}∼N(\mu_{t}^{\left(i\right)},\xi_{t}^{\left(i\right)})$$

Update the weight of this pose particle:

(26) $$\omega_{t}^{\left(i\right)}=\omega_{t-1}^{\left(i\right)}\cdot \eta^{\left(i\right)}$$

(4) Second Update:

In the second update, use the KFUWB value $s_{t}^{KFUWB}$ to correct the mean and variance of each particle. Assume the location information of KFUWB at time t is:

(27) $$s_{t}^{KFUWB}∼N(\mu_{t}^{KFUWB},\sigma_{t}^{KFUWB^{2}})$$

where $s_{t}^{KFUWB}={\left[x_{t}^{KFUWB},y_{t}^{KFUWB}\right]}^{T}$; $\mu_{t}^{KFUWB}$, $\sigma_{t}^{KFUWB^{2}}$ are the mean and variance of KFUWB at time t. $s_{t}^{KFUWB}$ does not include azimuth information, so only the particle’s position is corrected using KFUWB information, and the attitude information remains unchanged. Use Gaussian multiplication to correct the position information of each particle $s_{t}^{\left(i\right)}∼N(\mu_{t}^{\left(i\right)},\xi_{t}^{{\left(i\right)}^{2}})$ [55]:

(28) $$\mu_{t}^{\prime (i)}=\frac{\xi_{t}^{{\left(i\right)}^{2}}\mu_{t}^{KFUWB}+\sigma_{t}^{KFUWB^{2}}\mu_{t}^{\left(i\right)}}{\xi_{t}^{\left(i\right)}+\sigma_{t}^{KFUWB}}$$

(29) $$\xi_{t}^{\prime {\left(i\right)}^{2}}=\frac{\xi_{t}^{{\left(i\right)}^{2}}\delta_{t}^{KFUWB^{2}}}{\xi_{t}^{{\left(i\right)}^{2}}+\sigma_{t}^{KFUWB^{2}}}$$

In this way, each particle contains the position information of KFUWB. The corrected mean and variance are taken as the new particle position distribution information, denoted as $s_{t}^{\left(i\right)}∼N(\mu_{t}^{\prime (i)},\xi_{t}^{\prime {\left(i\right)}^{2}})$. Subsequently, a resampling step is performed to validate the particles.

(5) Resampling:

Calculate the effective sample size and judge whether resampling needs to be performed, and filter particles according to the weights of all particles [56]. Particles with higher weights are closer to the real attitude. The threshold T for the number of effective particles is set to 20.

(30) $$N_{eff}=\frac{1}{1+\sum_{i=1}^{k}{\left(\omega_{t}^{\left(i\right)}\right)}^{2}}$$

If $N_{eff}$ is less than the threshold T, perform the resampling operation.

(6) Pseudo-measurement:

Finally, the current position positioning result is obtained by the weighted average of the particle weights and the means of each particle [57]:

(31) $$s_{t}=\sum_{i=1}^{k}\mu_{t}^{\prime (i)}\omega_{t}^{\left(i\right)}$$

The obtained current pose information is used as the update value for the next step of extended Kalman filtering.

#### 2.3.3. Fusion of UWB/LiDAR/ODOM Based on Extended Kalman Filtering

**Figure 10:**
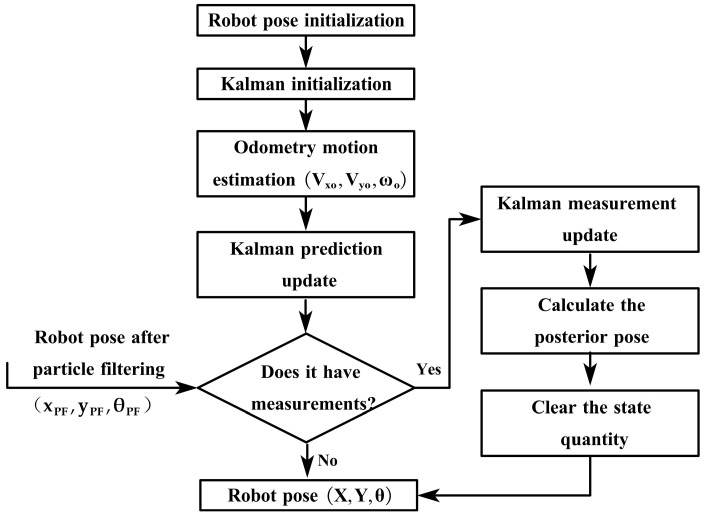
Flowchart of the EKF fusion process.

The EKF algorithm is proven to be effective in handling nonlinear systems, making it an ideal choice for integrating multiple sensor inputs and estimating the relative pose of robots [58]. We employ the EKF approach to fuse the odometry measurements and the pseudo-measurements obtained from the output of the PF to estimate the pose of the robot. The flowchart of the fusion algorithm is illustrated in Figure 10.

First, we use the pose information from the previous moment to initialize the robot’s pose, and the covariance matrix of the filter is initialized based on prior research experience. Next, the pose information $u={\left[V_{x,o},V_{y,o},\omega_{o}\right]}^{T}$ obtained from the odometer is used as the control input for the prediction phase. Then, the ${\left[x_{PF},y_{PF},\theta_{PF}\right]}^{T}$ obtained from the particle filtering fusion in the previous phase is used as the system state measurement input. Finally, the system state vector $x={\left[X,Y,\theta \right]}^{T}$ is calculated using the input state measurement value ${\left[x_{PF},y_{PF},\theta_{PF}\right]}^{T}$ and the Kalman gain coefficient $K_{t}$.

(1) Definition of System Dynamic Equation and Measurement Equation:

Assuming the mobile robot’s workspace in a trellis-style kiwifruit orchard as a two-dimensional environment, then the system state vector is the robot’s pose, and the robot platform’s state vector is $x={\left[X,Y,\theta \right]}^{T}$. Using the EKF algorithm, establish the dynamic equation and measurement equation of the motion system as follows [59].

System Dynamic Equation:

(32) x(t)=f(x(t−1),u(t),w(t))

System Measurement Equation:

(33) z(t)=h(x(t),v(t))

w and v follow a Gaussian distribution with a mean of 0. They are characterized by the probability distributions p(w)∼N(0,Q) and p(v)∼N(0,R).

(2) The odometry prediction model:

The input $u_{t}=\left[V_{x,ot},V_{y,ot},\omega_{ot}\right]$ of the odometer at time t is used as the control input for the prediction phase. Then, according to the method of dead reckoning and the motion model of the mobile robot [60], the robot’s pose at time t is expressed as:

(34) $$x_{t}^{-}=x_{t-1}^{}+\left(\begin{array}{ccc} \cos\theta_{t-1} & -\sin\theta_{t-1} & 0\\ \sin\theta_{t-1} & \cos\theta_{t-1} & 0\\ 0 & 0 & 1 \end{array}\right)\left(\begin{array}{l} V_{x,ot}\\ V_{y,ot}\\ \omega_{ot} \end{array}\right)dt+wdt$$

At the prediction stage, the covariance matrix of the system state vector at time t is written as:

(35) $$P_{t}^{-}=F_{}P_{t-1}F_{}{}^{T}+Q$$

The state transition matrix F and process noise covariance matrix Q can be calculated according to the odometer prediction model.

(3) Update Phase:

The ${\left[x_{PFt},y_{PFt},\theta_{PFt}\right]}^{T}$ obtained by PF fusion at time t is used as the system measurement value $z_{t}$ at time t. The measurement model is expressed as:

(36) $$z_{t}=\left(\begin{array}{l} x_{PFt}\\ y_{PFt}\\ \theta_{PFt} \end{array}\right)+v_{t}$$

Calculate the Kalman gain,$K_{t}$:

(37) $$K_{t}=P_{t}^{-}H^{T}{\left(HP_{t}^{-}H^{T}+R\right)}^{-1}$$

R is determined by considering the covariance matrices provided by LiDAR and UWB manufacturers.

Calculate the corrected state quantity and the corrected covariance matrix at time t

(38) $$x_{t}=x_{t}^{-}+K_{t}\left[z_{t}-x_{t}^{-}\right]$$

(39) $$P_{t}=(I-K_{t}H)P_{t}^{-}$$

## 3. Simulation and Experiments

### 3.1. Positioning Experiments in a Simulated Environment

**Figure 11:**
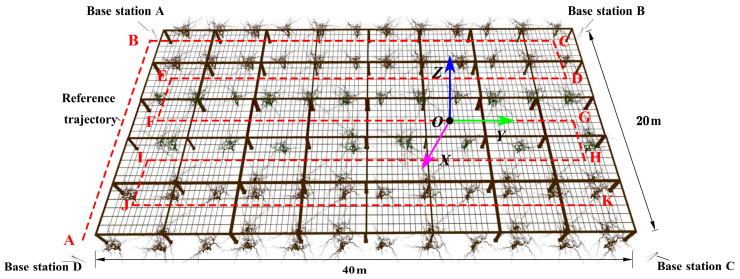
Experimental site layout.

To validate the effectiveness of the proposed mobile robot positioning algorithm, we conducted simulation experiments in a trellis-style kiwifruit orchard environment using the Gazebo platform. First, as illustrated in Figure 11, we constructed a trellis-style kiwifruit orchard model within the Gazebo environment that had dimensions of 40 m × 20 m × 1.8 m. The inter-row of the actual trellis-style kiwifruit orchard is a loose soil road surface, the soil type is mainly red clay, the parent material of this soil is loess, 0~100 cm is long-term cultivated by humans; 100~200 cm soil texture is uniform, mainly silty clay loam, soil bulk density 1.28 g/cm^3^ [61]. While building the ground model, we simulated the undulation of the terrain. The softness of the ground was simulated by adjusting the elastic parameters and friction parameters related to the ground in Gazebo. When the crawler travels on a loess road surface, it necessitates the construction of a three-dimensional model through Bekker’s proposed caterpillar–soil interaction model and determining the three-dimensional model through reasonable assessment [62,63]. However, as Gazebo cannot provide such a force mode, we assumed the ground to be hard during the simulation process. The interaction between the soil and the crawler track was achieved through a spring-damping model. Figure 11 includes four UWB base stations, trellis columns, trellis wire mesh, and kiwifruit trunks on both sides of the trellis, where the dashed trajectory A→B→C→D→E→F→G→H→I→J→K is the simulated operation path of the robot. Secondly, we set up UWB base stations. The four UWB base stations were arranged at the four vertices of the area, with Base Station A coordinates (−11, −26, 0), Base Station B coordinates (−11, 16, 0), Base Station C coordinates (11, 16, 0), and base station D coordinates (11, −26, 0). Finally, we controlled the robot’s movement in the field and used the SLAM algorithm to establish a 2D grid map required for robot localization.

In this study, we use the RMSE to evaluate the lateral trajectory error [64], which is defined as follows:

(40) $$RMSE=\sqrt{\frac{1}{n}\sum_{i=1}^{n}{\left({\hat{y}}_{i}-y_{i}\right)}^{2}}$$

where n represents the total number of samples, ${\hat{y}}_{i}$ represents the measured values of each sample data, and $y_{i}$ represents the reference values of each sample data.

#### 3.1.1. UWB Positioning Experiment

The factors that affect UWB positioning error are primarily related to sensor noise and obstacle noise. For instance, structural obstacles such as wires, branches, and leaves can cause signal attenuation and lead to ranging biases (non-line-of-sight errors), thereby affecting the final positioning accuracy. In this study, we simulated the influence of obstacles in complex environments by introducing ranging errors for each base station to evaluate the impact of non-line-of-sight errors on positioning accuracy. Additionally, we added Gaussian noise to the distance information between the tag and the four base stations to assess the effect of sensor noise on positioning accuracy. After we initialized the robot’s pose, we controlled the robot platform using the navigation package in ROS to autonomously navigate along the trajectory A→B→C→D→E→F→G→H→I→J→K at a speed of 0.5 m/s, as shown in Figure 11. The UWB positioning accuracy test results are presented in Table 1.

When we added Gaussian noise of (0, 0.1), and the range error for the base stations was 0 m, indicating no influence from environmental obstacles, the positioning error was minimal, measuring only 0.13 m. However, if there was an obstruction-induced increase in the range distance for one of the base stations, such as Base Station A (ranging from 0.3–1.0 m), the error increased from 0.25 m to 0.67 m accordingly. Similarly, if the range distances for two base stations increased due to obstruction, such as a 0.5 m error for Base Station A and an increase from 0.5 m to 1.0 m for Base Station B, the corresponding positioning error increased from 0.43 m to 0.82 m. The maximum positioning error occurred when the range distances for three base stations increased due to obstruction, with a 0.5 m error for Base Station A, a 0.5 m error for Base Station B, and a 1.0 m error for Base Station C. When we added Gaussian noise of (0, 0.3), the corresponding positioning error increased further. Furthermore, there was a significant difference between the root mean square error and the maximum positioning error, indicating the presence of fluctuations and discontinuities in UWB positioning accuracy. If the UWB positioning data were directly used as input for integrated positioning, it could introduce an additional offset and degrade fusion performance. We presented the positioning accuracy after UWB Kalman filtering in Table 2.

After we applied Kalman filtering, in various scenarios with increasing environmental noise and sensor noise, the maximum lateral positioning error of UWB positioning was reduced by an average of 50.1%, and the root mean square lateral error was reduced by an average of 31.1%. This demonstrates that the dynamic error correction method based on Kalman filtering can effectively improve the accuracy of UWB positioning.

#### 3.1.2. Experimental Evaluation of Trajectory Tracking Positioning

**Figure 12:**
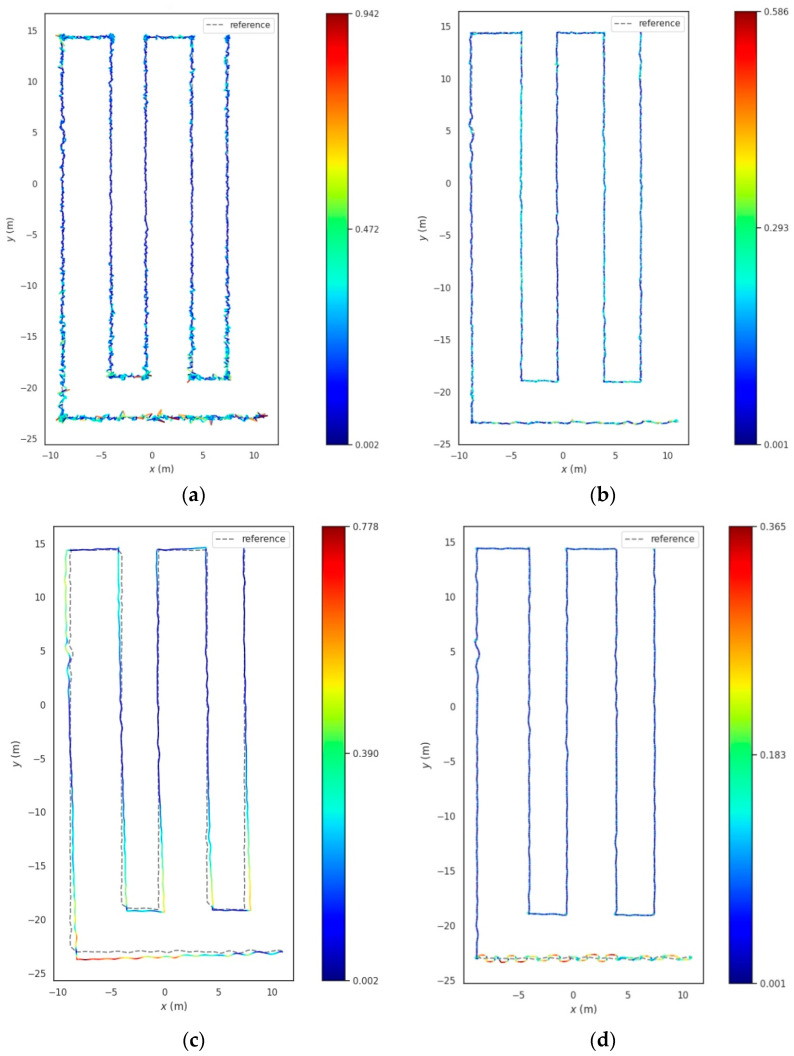
The positioning results under different positioning methods. (**a**) UWB. (**b**) KFUWB. (**c**) LiDAR/ODOM. (**d**) UWB/LiDAR/ODOM.

In the trellis-style kiwifruit orchard, signal propagation between the base stations is primarily hindered by trellis poles and tree branches, amongst other minor obstructions. To simulate the effect of orchard obstruction on UWB positioning in real-world conditions, we selected Base Station A with a ranging error of 0.1 m, Base Station B with a ranging error of 0.1 m, and Base Station C with a ranging error of 0.1 m. The robot was autonomously navigated at a speed of 0.5 m/s along the trajectory A→B→C→D→E→F→G→H→I→J→K, as shown in Figure 11, under different positioning scenarios including UWB standalone positioning, UWB Kalman filtering standalone positioning, LiDAR/ODOM positioning, and UWB/LiDAR/ODOM integrated positioning. We presented the obtained results in Figure 12.

**Figure 13:**
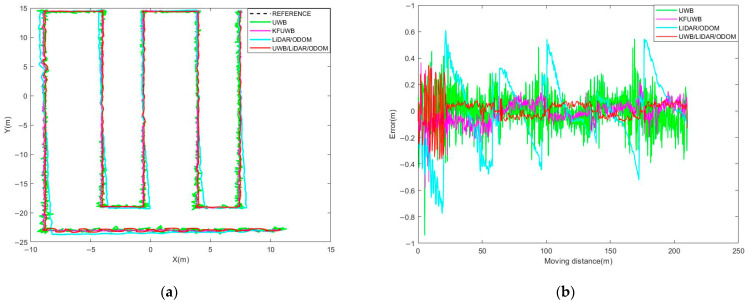
Comparison of trajectories for different positioning methods. (**a**) Trajectories. (**b**) Lateral error.

According to Figure 12a, it could be observed that under UWB standalone positioning, the robot’s positioning trajectory, represented by scattered points, was distributed around the reference trajectory. The positioning results were relatively scattered, indicating susceptibility to interference, with a maximum error of up to 0.942 m. In Figure 12b, under UWB Kalman filtering standalone positioning, the robot’s positioning results showed some improvement compared to the previous case, but the positioning was discontinuous and exhibited poor resistance to interference, with a maximum error of 0.586 m. Figure 12c shows the case of LiDAR/ODOM integrated positioning. In the AB segment, where the robot transitioned from the ground to the trellis structure, the positioning performance was significantly worse due to the lack of features on one side of the environment. This resulted in a significant offset, and the positioning error after point B mainly accumulated and increased over time. In Figure 12d, under UWB/LiDAR/ODOM integrated positioning, the indistinct features in the AB segment adversely affected the performance of LiDAR/ODOM positioning, leading to larger oscillations in the UWB/LiDAR/ODOM integrated positioning. However, compared to LiDAR/ODOM positioning, there was a considerable improvement. After point B, the robot’s trajectory was smoother and the cumulative error was reduced, indicating improved positioning performance. The lateral errors between the recorded trajectory and the reference trajectory were compared, as shown in Figure 13. A summary of the statistical analysis and calculations of the lateral errors for each positioning method can be found in Table 3.

The average lateral error of the proposed UWB/ODOM/LiDAR integrated positioning method in this study was 0.076 m, with a maximum error of less than 0.4 m. Compared to the UWB positioning, KFUWB positioning, and LiDAR/ODOM integrated positioning methods, the average positioning error was reduced by 55.3%, 44.5%, and 67.7%, respectively. The RMSE was reduced by 53.9%, 37.9%, and 66.0%. It can be concluded that the proposed integrated positioning method in this study exhibits superior positioning performance compared to the other three positioning methods.

#### 3.1.3. Target Points Positioning Experiment

**Figure 14:**
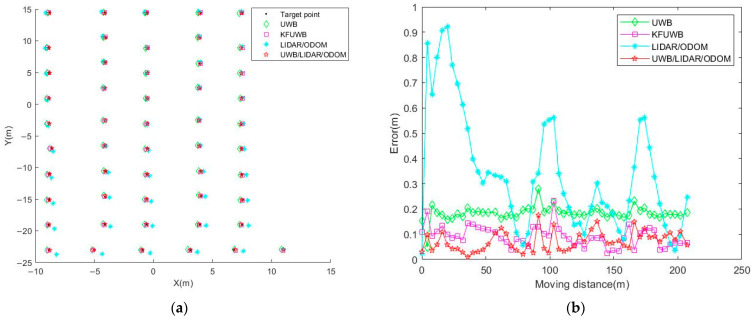
Comparison of target point localization accuracy among different positioning methods. (**a**) Positioning results. (**b**) Positioning error.

We set fifty-one target points along the trajectory A→B→C→D→E→F→G→H→I→J→K, as shown in Figure 11, for multi-target point autonomous navigation. When the robot reached each target point, it paused for 2 min to record the positioning data using different positioning methods at that specific moment, and we computed the average values. The positioning results are presented in Figure 14.

Significant positioning errors might be exhibited by the LiDAR/ODOM integrated positioning method due to the ambiguity of positioning features, and it could also suffer from large positioning errors due to cumulative errors in environments with distinct positioning features. In contrast, the UWB and KFUWB methods demonstrate higher accuracy in target point positioning when the positioning was performed in a static state. The integrated UWB/LiDAR/ODOM positioning method outperformed the other three methods and provided the most accurate positioning results. The analysis of the experimental data for target point positioning is summarized in Table 4.

The largest source of positioning error in LiDAR/ODOM integrated positioning was due to the fact that there were positioning features on only one side of the robot during the simulation path segment AB. This resulted in noticeably poorer positioning performance at target points in the AB segment, thereby leading to a larger overall positioning error. The non-line-of-sight error in UWB solo positioning was significantly higher than in KFUWB solo positioning and integrated positioning. UWB/LiDAR/ODOM integrated positioning first used KFUWB and LiDAR/ODOM to perform PF fusion and then fused with the ODOM value through extended Kalman filtering. Thus, its positioning was more continuous than KFUWB and did not experience large fluctuations; moreover, it used the KFUWB value to suppress the cumulative error of LiDAR/ODOM during the fusion process. Hence, as could be seen from the table, compared to UWB positioning, KFUWB positioning, and LiDAR/ODOM integrated positioning, the overall positioning accuracy of the UWB/LiDAR/ODOM integrated positioning method had improved by 60.8%, 21.7%, and 79.6%, respectively. The RMSE of positioning on the x-axis, y-axis, and overall was 0.047, 0.046, and 0.072 m, respectively, with the maximum positioning error being 0.174 m. These results demonstrated that the positioning method adopted in this study improved the precision of the robot.

### 3.2. Positioning Experiments in a Kiwifruit Orchard Environment

**Figure 15:**
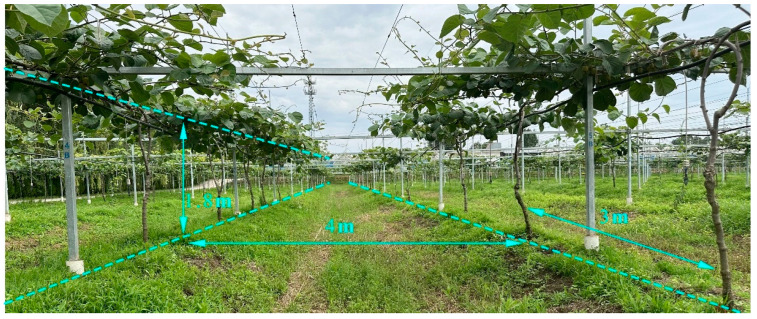
Kiwifruit orchard environment field.

**Figure 16:**
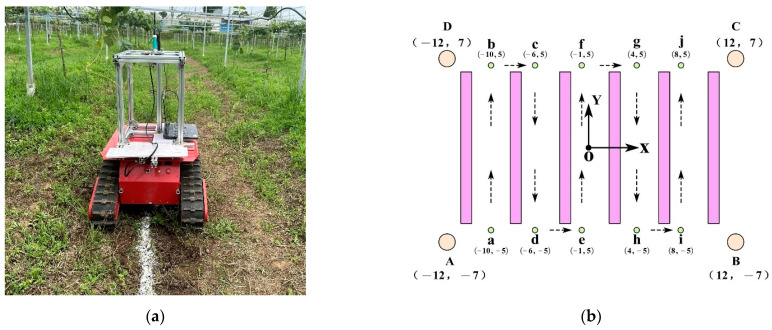
Positioning experiment. (**a**) Positioning experiment scene image. (**b**) Positioning experiment route diagram.

We conducted positioning experiments in a Kiwifruit Orchard Environment at the Yangling International Kiwifruit Innovation and Entrepreneurship Park (34°18′21″ N, 108°3′41″ E), as shown in Figure 15. The orchard, cultivated using a trellis system, features a row spacing of 4 m, column spacing of 2 m, a canopy height of 1.8 m, and covers a total area of 336 m^2^. We equipped the crawler robot, depicted in Figure 16a, with odometry, LiDAR, UWB tags, and a tracking device. Real-time information is collected using odometry and LiDAR, and the cartographer algorithm is employed to construct a two-dimensional grid map of the kiwifruit orchard [65,66]. In Figure 16b, A, B, C, and D represent the four UWB base stations deployed in the field. We used a laser rangefinder, with an accuracy of 1 mm, to obtain distance measurements. During the experiment, the robot was controlled to initiate its movement from point a and follow the sequence of a→b→c→d→e→f→g→h→i→j. A tracking device containing white flour was placed on the robot, and as it traversed the designated path, the white line left on the ground represents the actual trajectory followed by the robot. A comparison was then made between the positioning results obtained through sensor measurements and the actual path taken by the robot to assess the disparities.

**Figure 17:**
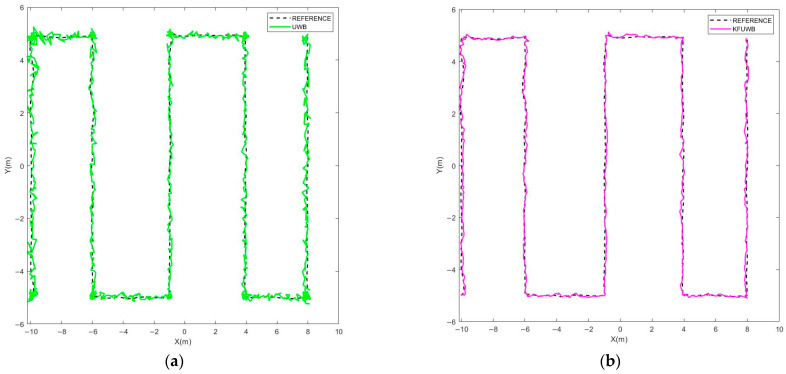

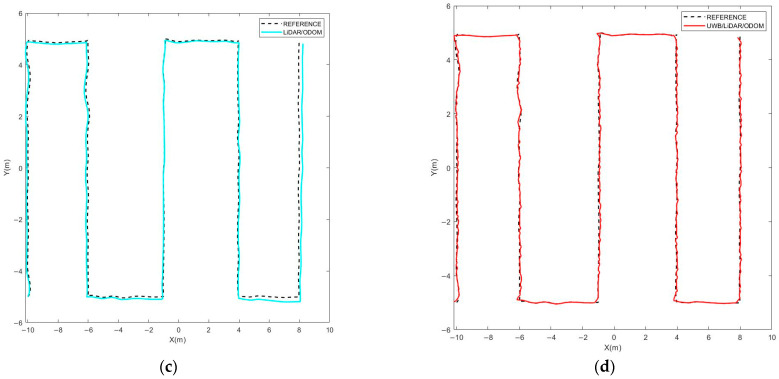
Positioning trajectories of different positioning methods. (**a**) UWB. (**b**) KFUWB. (**c**) LiDAR/ODOM. (**d**) UWB/LiDAR/ODOM.

**Figure 18:**
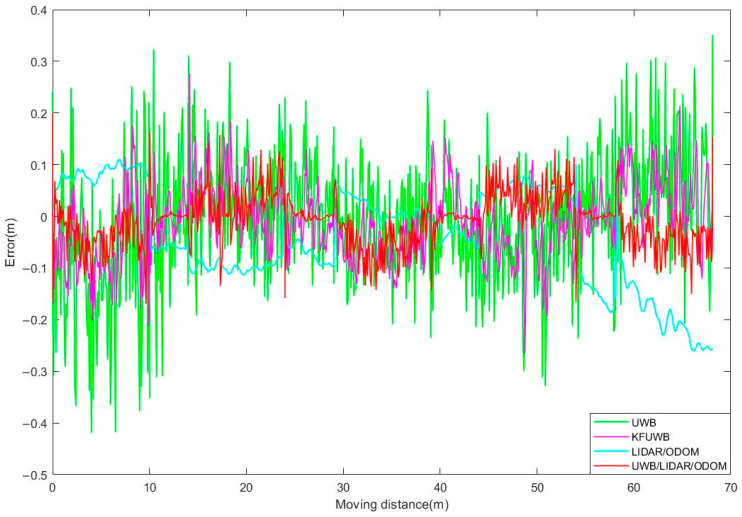
Lateral errors of different positioning methods.

We manually controlled the robot at a speed of 0.5 m/s along the sequence shown in Figure 16b, under different positioning scenarios including UWB standalone positioning, UWB Kalman filtering standalone positioning, LiDAR/ODOM positioning, and UWB/LiDAR/ODOM integrated positioning. The obtained results are presented in Figure 17 and Figure 18.

In Figure 17, the dashed line corresponds to the robot’s actual path. In Figure 17a, the green trajectory represents UWB standalone localization, while in Figure 17b, the pink trajectory represents UWB Kalman filtering standalone localization. In Figure 17c, the light blue trajectory represents LiDAR/ODOM integrated localization, and in Figure 17d, the red trajectory represents UWB/LiDAR/ODOM integrated localization.

Observations from Figure 17 and Figure 18 reveal that in the case of UWB standalone positioning, the robot’s positioning trajectory is fairly scattered around the reference trajectory. A noticeable improvement in the positioning results was observed when employing UWB Kalman filtering standalone positioning. Under LiDAR/ODOM integrated positioning, the initial positioning error was small, but it gradually increased over time, resulting in continuous positioning. Under UWB/LiDAR/ODOM integrated positioning, the robot’s positioning trajectory closely followed the reference trajectory with minimal deviation, resulting in continuous and the most accurate positioning. A summary of the positioning results can be found in Table 5.

For UWB positioning, the average error was 0.092 m, with a maximum error of 0.419 m and an RMSE of 0.142 m. For UWB Kalman filtering positioning, the maximum error was 0.276 m, with a lateral RMSE of 0.058. The LiDAR/ODOM integrated positioning method had an average error of 0.084 m, a maximum lateral error of 0.260 m, and an RMSE of 0.081 m. The proposed UWB/LiDAR/ODOM integrated positioning method in this study had an average lateral error of 0.044 m, with a maximum error of less than 0.2 m. Compared to UWB positioning, UWB Kalman filtering, and LiDAR/ODOM integrated positioning methods, the lateral RMSE was reduced by 64.8%, 13.8%, and 38.3%, respectively. The experimental results demonstrated that the proposed integrated positioning method exhibits better adaptability and positioning performance in the kiwifruit orchard environment.

## 4. Discussion

(1) When the mobile robot traveled between rows in the kiwifruit orchard at a low speed, the inertia during the robot’s deceleration could be significant, thereby affecting the tracking error of the autonomous navigation system. Additionally, when the robot moved at a high speed, the central control unit might not have been able to provide accurate positioning information for the transport robot. Based on preliminary real-world tests, we set the robot’s travel speed to 0.5 m/s in the experimental process.
(2) Based on the results obtained from both simulation and real-world experiments, it could be concluded that the positioning trajectories of UWB and KFUWB, as absolute positioning methods, exhibited a discrete nature. On the other hand, the positioning trajectories of LiDAR/ODOM and UWB/LiDAR/ODOM, as integrated positioning methods, appeared to be continuous. This distinction arises from the fact that absolute positioning methods do not rely on the previous positioning results but are influenced only by the sensor and environmental noise. The independent use of UWB and KFUWB for navigation and positioning may potentially affect the stability and reliability of robot navigation.
(3) The simulation experiment results indicated that LiDAR/ODOM, as an integrated positioning method, exhibits the largest lateral error among the four positioning methods. However, in real-world experiments, UWB standalone positioning demonstrates the largest lateral error among the four methods. This discrepancy may be attributed to the fact that the simulated operation path included a stage where the robot transitioned from the field to the canopy (AB segment). During this AB segment, the robot had limited positioning features on only one side, resulting in poorer positioning performance. Consequently, this led to certain deviations in the positioning trajectory beyond point B. In real-world experiments, the robot directly entered the canopy, where the LiDAR/ODOM positioning features were prominent, resulting in relatively continuous positioning results. Additionally, environmental disturbances significantly affected UWB positioning, thus explaining the largest UWB positioning error among the four methods.
(4) This study had two main contributions. First, it proposes a Kalman filter-based dynamic UWB error correction method that is applicable to trellised kiwifruit orchards. Secondly, this study proposes an integrated positioning method based on UWB/LiDAR/ODOM and conducts extensive tests in simulated and real environments to evaluate the feasibility of the positioning method. The integrated positioning method of this study is different from using EKF alone for sensor information fusion or using PF alone for sensor information fusion. Instead, the integrated positioning method of this study is implemented by combining EKF and PF. EKF is used for sensor fusion and combines with PF for measurement updates. The combination of EKF and PF can provide better accuracy than using either filter separately [67,68]. This is primarily because each filter can compensate for the weaknesses of the other, thereby reducing errors in the position update process. EKF uses a Gaussian noise model, which may be inaccurate for non-linear systems, while PF does not assume the nature of the noise and is more flexible. In this study, when there are multiple credible explanations for sensor data, the method of combining EKF-PF will be more effective and more suitable for the environment of this study.
(5) Our integrated positioning method takes advantage of three fundamental filtering techniques: KF, PF, and EKF. Each of these methods has its unique mathematical basis and, when combined, they can effectively merge data from multiple sensors to deliver improved results. When these techniques are combined, the integrated positioning method initially applies the KF to UWB measurement values to reduce noise and enhance the accuracy of these measurements. Then, a PF works on LiDAR data, odometer readings, and Kalman-filtered UWB data, effectively integrating sensor data. Finally, the EKF combines the odometer readings with the posture estimated by the PF to provide the final optimal estimate of the robot’s posture. By integrating these filters and fully exploiting the advantages of each individual method, the overall method offers an optimized solution for multi-modal data fusion, particularly applicable to complex scenarios such as applications in trellis-style kiwifruit orchards.
(6) The simulation environment in this study has the following limitations. Firstly, the lack of simulation of certain natural conditions; in this study, the obstacles, terrain, and their physical properties in the kiwifruit orchard simulation environment are created based on real trellis-style orchards. However, all actual factors cannot be reproduced in a simulation environment, such as changes in lighting, climate conditions, and random interference, which could lead to deviations between simulation results and reality. Secondly, model errors: simulation models, being based on theories and mathematical formulas, may not precisely simulate complex physical and environmental attributes in the real world. This discrepancy might result in the integrated positioning method performing well in the simulation environment but underperforming actual applications. Then, sensor model limitations: sensor models used in the simulation environment may not accurately reflect the performance and characteristics of actual sensors, possibly impacting the performance of the sensor fusion positioning methods. Lastly, the gap between theory and practice: simulation usually relies on certain theoretical conditions, but these conditions may not be fully met in actual applications, leading to simulation results not accurately predicting the real situation. For example, actual sensors need to consider calibration and bias issues. While the simulation environment currently does not mimic all aspects of the real-world environment, our simulation model holds a certain degree of similarity compared to the real environment, making it a valid tool for verifying the effectiveness and applicability of the proposed integrated positioning method in kiwifruit orchard environments. Future work will further validate the algorithm proposed in this study in actual orchards.

To reduce the lateral deviation of the robot during turning, future research will focus on optimizing the pure tracking algorithm. Based on the experimental results, the design of fuzzy PID control rules for lateral deviation adjustment will be explored. This approach will utilize the derived rules and the pure tracking algorithm to enable the robot to adjust its turning radius based on changes in distance.

## 5. Conclusions

(1) This study proposed a UWB dynamic error correction method based on Kalman filtering. Twenty scenarios ranging from no obstruction to gradually increasing obstructions were simulated. By analyzing the positioning results before and after filtering, it was determined that the average positioning accuracy was improved by 31.1% after filtering.
(2) This study proposed an integrated positioning method based on UWB/LiDAR/ODOM. This method fused the filtered UWB values, LiDAR measurements, and ODOM values using particle filtering. The robot’s estimated pose was obtained by using the particle-filtered robot pose as the measurement for extended Kalman filtering and the ODOM values as the prediction. Finally, the prediction and measurement were fused using extended Kalman filtering to obtain the robot’s estimated pose.
(3) Simulation results of the positioning experiments demonstrated that the integrated positioning method proposed in this study effectively reduced the cumulative errors produced by LiDAR/ODOM integrated positioning and provided smoother positioning trajectories compared to UWB standalone positioning and UWB Kalman filtering standalone positioning. Field positioning accuracy comparison experiments showed that the proposed integrated positioning method improved the robot’s positioning accuracy compared to UWB standalone positioning, UWB Kalman filtering positioning, and LiDAR/ODOM integrated positioning methods. This approach largely addressed the issue of low positioning accuracy of mobile robots in the trellis kiwifruit orchard environment.

## Figures and Tables

**Table 1:** Influence of non-line-of-sight error on positioning error.

Base Station Distance Error (m)	RMSE (m) Gaussian (0, 0.1)	MAX (m) Gaussian (0, 0.1)	RMSE (m) Gaussian (0, 0.3)	MAX (m) Gaussian (0, 0.3)
0	0.13	0.78	0.24	1.05
A 0.3	0.25	1.15	0.39	1.38
A 0.5	0.36	1.40	0.48	1.44
A 0.8	0.51	1.70	0.64	1.88
A 1.0	0.67	2.00	0.79	2.11
A 0.5 + B 0.5	0.43	1.52	0.61	1.92
A 0.5 + B 0.8	0.60	1.90	0.84	2.20
A 0.5 + B 1.0	0.82	2.22	0.98	2.37
A 0.5 + B 0.5 + C 1.0	0.93	2.37	1.12	2.75
A 0.1 + B 0.1 + C 0.1	0.21	0.94	0.29	1.16

**Table 2:** Positioning error after Kalman filtering.

Base Station Distance Error (m)	RMSE (m) Gaussian (0, 0.1)	MAX (m) Gaussian (0, 0.1)	RMSE (m) Gaussian (0, 0.3)	MAX (m) Gaussian (0, 0.3)
0	0.11	0.40	0.13	0.53
A 0.3	0.15	0.58	0.19	0.71
A 0.5	0.21	0.83	0.31	0.85
A 0.8	0.30	0.84	0.40	0.89
A 1.0	0.45	0.92	0.56	0.95
A 0.5 + B 0.5	0.32	0.88	0.42	0.90
A 0.5 + B 0.8	0.47	0.94	0.63	1.01
A 0.5 + B 1.0	0.63	0.98	0.77	1.07
A 0.5 + B 0.5 + C 1.0	0.72	1.07	0.86	1.10
A 0.1 + B 0.1 + C 0.1	0.16	0.57	0.19	0.61

**Table 3:** Analysis of lateral positioning errors for different positioning methods.

Positioning Methods	Average Error (m)	Maximum Error (m)	Standard Deviation (m)	RMSE (m)
UWB	0.170	0.942	0.128	0.213
KFUWB	0.137	0.586	0.078	0.158
LiDAR/ODOM	0.235	0.778	0.168	0.289
UWB/LiDAR/ODOM	0.076	0.365	0.061	0.098

**Table 4:** Analysis of target point positioning errors under different positioning methods.

Positioning Methods	Average Error in the X-Direction (m)	Average Error in the Y-Direction (m)	Maximum Error (m)	RMSE (m)
UWB	0.175	0.035	0.280	0.184
KFUWB	0.059	0.061	0.232	0.092
LiDAR/ODOM	0.241	0.210	0.923	0.353
UWB/LiDAR/ODOM	0.047	0.046	0.174	0.072

**Table 5:** Analysis of lateral positioning errors for different positioning methods.

Positioning Methods	Average Error (m)	Maximum Error (m)	Standard Deviation (m)	RMSE (m)
UWB	0.092	0.419	0.078	0.142
KFUWB	0.051	0.276	0.042	0.058
LiDAR/ODOM	0.084	0.260	0.051	0.081
UWB/LiDAR/ODOM	0.044	0.199	0.033	0.050

## Data Availability

All data are presented in this article in the form of figures and tables.

## Appendix A

**Table A1:** Product specifications.

Product Categories	Product Models	Parameters	Specifications
LiDAR	RPLiDAR S1	Measurement radius	White Object: 40 m Black Object: 10 m
		Sampling rate	9200 times per second
		Scanning frequency	10 Hz
		Angular resolution	0.391°
		Communication rate	256,000 bps
		Range resolution	3 cm
		Measurement accuracy	±5 cm
		Application scenarios	Suitable for indoor and outdoor environments, reliable anti-glare capability
UWB	D-DWG-PGPLUS	Communication rate	115,200 bps
		Single communication	0.2 ms—6.8 M/s air speed
		Single-shot ranging	3 ms—6.8 M/s air speed
		Communication distance	600 m
		Communication frequency	3.5 Ghz–6.5 Ghz
		Measurement accuracy	±5 cm
Encoder	E6B2-CWZ6C	Resolution	360 pulses/rotation
		Maximum mechanical speed	6000 RPM
		Torque	Maximum 0.01 Nm
		Seismic resistance	Maximum 500 m/s²
		Rated voltage	5VDC
		Operating current	Maximum 40 mA
Motor	48V DC brushless motor	Rated power	1500 W
		Rated torque	144 NM

**Table A2:** Table of variable definitions.

Variable Types	Variable Symbols	Variable Meanings
Variables in the UWB Positioning Error Correction on KF.	$\left(x_{1},y_{1}\right)$	Robot position information measured by UWB at time $t_{1}$.
	$\left(x_{2},y_{2}\right)$	Robot position information measured by UWB at time $t_{2}$.
	$\left(x_{3},y_{3}\right)$	Robot position information predicted at time $t_{3}$.
	$\left(m_{3},n_{3}\right)$	Robot position information measured by UWB at time $t_{3}$.
	$z_{3}$	Robot position information measured by UWB at time $t_{3}$.
	$\left(x_{KFUWBt3},y_{KFUWBt3}\right)$	Robot position information fused by Kalman filtering at time $t_{3}$.
	$T_{f}$	Flight time of the measured signal.
	θ	Robot’s steering angle at time $t_{1}$.
	Δθ	Maximum value of angle deviation.
	N	Maximum value of distance deviation.
	$\left(x_{k},y_{k}\right)$	Robot’s position information at time k.
	$\left(x_{k-1},y_{k-1}\right)$	Robot’s position information at time k−1.
	$\left({\hat{x}}_{k},{\hat{y}}_{k}\right)$	Robot’s predicted position information at time k.
	A	State transition matrix.
	$u_{k}$	Input control vector at time k.
	B	Control matrix.
	$w_{k}$	Process noise.
	Q	Covariance matrix of the system process.
	$z_{k}$	Observation matrix at time k.
	$\left(z_{x},z_{y}\right)$	UWB measurement result at time k.
	H	Transformation matrix.
	$v_{k}$	Observation noise.
	R	Covariance matrix of observation noise.
	${\hat{X}}_{k}$	State prediction vector at time k.
	$X_{k}$	State vector at time k.
	$X_{k-1}$	State vector at time k−1.
	${\hat{P}}_{k}$	State prediction covariance matrix at time k.
	$P_{k}$	Covariance matrix at time k.
	$P_{k-1}$	Covariance matrix at time k−1.
	$K_{k}$	Kalman gain matrix at time k.
	I	Identity matrix.
Variables in Fusion of UWB/LiDAR/ODOM Based on Particle Filtering.	$C_{t-1}$	Particle swarm at time t−1.
	$C_{t-1}^{i}$	Particles at time t−1.
	$C_{t}$	Particle swarm at time t.
	$u_{t-1}$	Odometry measurement information at time t−1.
	$u_{t}$	Odometry measurement information at time t.
	$\left[V_{xt-1},V_{yt-1},\omega_{t-1}\right]$	Odometry measurement information at time t−1.
	$z_{t}$	LiDAR scan information at time t.
	$\left[x_{Lt},y_{Lt},\theta_{Lt}\right]$	LiDAR scan information at time t.
	${\hat{s}}_{t}$	Particle pose information at time t.
	$s_{t-1}$	Pose information at time t−1.
	$s_{t-1}^{i}$	Particle pose information at time t−1.
	$s_{t-1}^{\left(i\right)}$	Particle pose information at time t−1.
	$s_{t}$	Pose information at time t.
	$s_{t}^{\left(i\right)}$	Particle pose information at time t.
	$w_{t}$	Weight information at time t.
	$w_{t}^{\left(i\right)}$	Particle weight information at time t.
	$w_{t-1}$	Weight information at time t−1.
	$w_{t-1}^{i}$	Particle weight information at time t−1.
	$s_{t}^{\prime i}$	Estimated pose of particles at time t.
	${\hat{s}}_{t}^{i}$	Local extrema of particles at time t.
	$s_{t}^{KFUWB}$	Value of KFUWB at time t.
	$\left(x_{t}^{KFUWB},y_{t}^{KFUWB}\right)$	Value of KFUWB at time t.
	$\mu_{t}^{KFUWB}$	Mean of KFUWB at time t.
	$\sigma_{t}^{KFUWB^{2}}$	Variance of KFUWB at time t.
	$\mu_{t}^{\left(i\right)}$	Mean of particles at time t.
	$\xi_{t}^{\left(i\right)}$	Variance of particles at time t.
	$\mu_{t}^{\prime (i)}$	Mean of particles after correction at time t.
	$\xi_{t}^{\prime {\left(i\right)}^{2}}$	Variance of particles after correction at time t.
	T	Threshold of effective particle number.
	$N_{eff}$	Effective sample capacity.
Variables in Fusion ofUWB/LiDAR/ODOM Based on Extended Kalman Filtering.	u	Odometry measurement information.
	$u_{t}$	Odometry measurement information at time t.
	$\left(V_{x,o},V_{y,o},\omega_{o}\right)$	Odometry measurement information.
	$V_{x,ot}$	Linear velocity of the robot in the x-direction as measured by odometry at time t.
	$V_{y,ot}$	Linear velocity of the robot in the y-direction as measured by odometry at time t.
	$\omega_{ot}$	Angular velocity of the robot as measured by odometry at time t.
	$\left(x_{PF},y_{PF},\theta_{PF}\right)$	Fused value from particle filtering.
	$\left(x_{PFt},y_{PFt}\right)$	Robot’s global position in the global coordinate system fused by particle filtering at time t.
	$\theta_{PFt}$	Robot’s global yaw angle fused by particle filtering at time t.
	$K_{t}$	Kalman gain coefficients.
	x	System state vector.
	$x_{t-1}^{}$	State vector at time t−1.
	$x_{t}^{-}$	State prediction vector at time t−1.
	(X,Y)	Robot’s global position in the global coordinate system.
	θ	Robot’s orientation angle.
	w	Process noise.
	v	Observation noise.
	Q	Covariance matrix of noise.
	R	Covariance matrix of observation noise.
	$P_{t}^{-}$	Covariance prediction matrix at time t.
	$P_{t}$	Covariance matrix at time t.
	$P_{t-1}$	Covariance matrix at time t−1.
	F	State transition matrix.
	$z_{t}$	Observation matrix at time t.
	H	Jacobian matrix of the measurement model.
	I	Identity matrix.
